# Triple-Negative Apocrine Carcinoma: Largest Cohort Highlights Unique Biology and Survival Advantage

**DOI:** 10.3390/jcm14197103

**Published:** 2025-10-09

**Authors:** Tugba Basoglu, Ugur Ozkerim, Sila Oksuz, Oguzcan Kinikoglu, Sedat Yildirim, Sermin Kokten, Heves Surmeli, Deniz Isik, Ozlem Nuray Sever, Seval Ay Ersoy, Hatice Odabas, Nedim Turan

**Affiliations:** 1 Department of Medical Oncology, Kartal Dr. Lütfi Kirdar City Hospital, Health Science University, 34865 Istanbul, Turkey; ugur.ozkerim@hotmail.com (U.O.); sila.oksuz@gmail.com (S.O.); ogokinikoglu@yahoo.com (O.K.); rezansedat@hotmail.com (S.Y.); hevessurmeli@hotmail.com (H.S.); dnz.1984@yahoo.com (D.I.); drsevalay@gmail.com (S.A.E.); odabashatice@yahoo.com (H.O.); turan.nedim@hotmail.com (N.T.); 2 Department of Pathology, Kartal Dr. Lutfi Kirdar City Hospital, Health Science University, 34865 Istanbul, Turkey; serminkokten@gmail.com; 3 Department of Medical Oncology, Sancaktepe Sehit Prof. Dr. Ilhan Varank City Hospital, Health Science University, 34785 Istanbul, Turkey; ozlem.sever@hotmail.com

**Keywords:** triple-negative breast cancer, apocrine carcinoma, androgen receptor, neoadjuvant chemotherapy, pathological complete response, Ki-67, delta Ki-67, tumor-infiltrating lymphocytes, survival outcomes, breast cancer subtypes

## Abstract

**Background/Objectives**: Triple-negative breast cancer (TNBC) is a heterogeneous entity lacking ER, PR, and HER2, with aggressive biology and high recurrence risk. Neoadjuvant chemotherapy (NACT) is the standard of care, and a pathological complete response (pCR) is a surrogate marker for survival. Within TNBC, apocrine differentiation (TNAC) is a distinct subtype, often androgen receptor (AR)-positive, with lower chemosensitivity but a favorable prognosis. Comparative studies of TNAC versus classical TNBC remain limited. This study aimed to define clinical and biological differences between TNAC and non-apocrine TNBC (NA-TNBC), representing the largest TNAC cohort to date. **Methods**: This retrospective study included 129 non-metastatic TNBC patients treated with NACT and surgery (2010–2020). Patients were classified as TNAC or NA-TNBC. Demographic, clinicopathological, and immunohistochemical data (including Ki-67 and AR) were collected. Tumor-infiltrating lymphocytes (TILs), delta Ki-67, pathological complete response (pCR), and survival outcomes were evaluated. **Results**: Of 129 TNBC patients, 45 (34.9%) were TNAC. AR positivity occurred in 64.4% of TNACs. TNAC patients were predominantly postmenopausal. pCR rates were significantly lower in TNAC (6.6% vs. 30.9%, *p* = 0.002). TNACs exhibited lower baseline Ki-67, delta Ki-67, and TIL positivity (13.3% vs. 30%). Despite this, 5-year overall survival was higher in TNAC (86% vs. 78%). Delta Ki-67 > 20% strongly predicted pCR across the cohort (*p* < 0.001). Carboplatin was rarely used in TNAC (8.3%), but was associated with a higher pCR rate (50% vs. 2.4%, *p* = 0.018). **Conclusions**: TNAC represents a biologically distinct TNBC subtype, characterized by low pCR but favorable survival. Recognition of its unique features may guide treatment de-escalation and exploration of AR-targeted therapies. Prospective studies focusing on TNAC are warranted.

## 1. Introduction

Triple-negative breast cancer (TNBC) accounts for approximately 15–20% of all breast carcinomas and is defined immunohistochemically by the absence of ER, PR, and HER2 expression [1,2]. This phenotype excludes patients from receiving endocrine or HER2-targeted therapies, leaving cytotoxic chemotherapy as the principal systemic treatment modality. Clinically, TNBC is characterized by early onset, high histological grade, increased risk of visceral metastasis, and poor overall survival compared to hormone receptor-positive or HER2-amplified subtypes [3,4].

NACT is frequently employed in TNBC management for tumors > 2 cm or node-positive disease, with pCR serving as a surrogate marker for improved disease-free and overall survival [5]. Nevertheless, TNBC is not a uniform entity. Molecular profiling has identified substantial heterogeneity within TNBC, leading to the classification of distinct subtypes such as basal-like (BL), mesenchymal (M), immunomodulatory (IM), and luminal androgen receptor (LAR) [6,7].

Recent advances in systemic therapy have further reshaped the management landscape of TNBC. In particular, perioperative immunotherapy combined with chemotherapy has emerged as a standard of care in high-risk, early-stage TNBC, as demonstrated by recent systematic reviews and network meta-analyses [8]. In parallel, comprehensive reviews have highlighted the expanding role of novel systemic therapies—including immune checkpoint inhibitors, PARP inhibitors, and antibody–drug conjugates—in improving outcomes for TNBC patients [9].

Among these, triple-negative apocrine carcinoma (TNAC) stands out as a histologically and biologically distinct subtype. TNAC is characterized by well-defined apocrine features—abundant eosinophilic cytoplasm, sharply demarcated cell borders, and prominent nucleoli—in addition to the triple-negative immunoprofile. Although apocrine carcinomas comprise only 1–4% of all breast cancers, around 20–30% of these are triple-negative, thereby constituting the TNAC subtype [10,11].

Molecularly, TNAC exhibits a “luminal apocrine” phenotype, marked by high AR expression (>90%), luminal markers (FOXA1, GATA3), and frequent alterations in the PI3K/AKT/mTOR pathway. These features contrast sharply with the genomic instability, basal cytokeratin expression, and BRCA mutations observed in classical TNBC subtypes [12,13,14,15]. This may explain the paradox of TNAC: despite low chemosensitivity, these tumors often exhibit more indolent progression and improved long-term outcomes [16,17,18,19].

Furthermore, the immunological profile of TNAC is unique. These tumors tend to be “immune-cold,” with lower levels of tumor-infiltrating lymphocytes (TILs), reduced CD8+ T cell infiltration, and limited PD-L1 expression—traits that contribute to resistance against immunotherapy [20,21,22].

Beyond immune-cell composition, the tumor microenvironment (TME) in TNBC is also shaped by tumor-derived exosomes that mediate intercellular communication with dual roles in tumor progression and immune regulation. These mechanisms are highly relevant to TNAC, which displays an immune-cold phenotype. Recent advances in systems approaches—network pharmacology and multi-omics—offer a framework to delineate pathway-level differences between TNAC and other TNBC subtypes, including AR/LAR-like programs, PI3K/AKT signaling, and p53 network cross-talk [23,24,25,26]. In particular, dysregulated microRNAs linked to the p53 signaling axis and the exploratory therapeutic potential of natural compounds such as resveratrol have been highlighted in TNBC biology [27,28], underscoring the need for mechanistic, biomarker-driven strategies in TNAC.

Despite increasing recognition of TNAC as a discrete biological entity, comparative clinical data regarding its response to NACT, immune landscape, and survival outcomes remain sparse. Most available data are derived from case series or molecular profiling studies without robust clinical correlation.

This study represents one of the largest cohorts of TNAC patients to date. It aims to systematically evaluate clinical characteristics, treatment response, survival outcomes, and the prognostic significance of AR and delta Ki-67. By contrasting TNAC with classical NA-TNBC, we provide new insights to support subtype-specific therapeutic approaches and inform future clinical trial designs.

## 2. Materials and Methods

This retrospective cohort study was approved by the Ethics Committee of the University of Health Sciences, Kartal Dr. Lütfi Kırdar Training and Research Hospital (Approval No: Approval No: 2025/010.99/15/13). Data were obtained from institutional records between January 2010 and January 2022.

### 2.1. Inclusion and Exclusion Criteria

Patients were eligible if they had:

- Histologically confirmed invasive ductal carcinoma with a triple-negative phenotype (ER-/PR-/HER2-);
- Completed standard NACT followed by surgery;
- Available pre- and post-treatment Ki-67 index;
- Complete demographic, pathological, and treatment records;
- Patients were excluded if they had;
- HER2-positive or hormone receptor-positive disease;
- Stage IV (metastatic) disease at diagnosis;
- Non-ductal histology or missing pathological data.

### 2.2. Histopathological Assessment

All hematoxylin and eosin (H&E)-stained sections were independently reviewed by two experienced breast pathologists. Tumors were classified as TNAC if ≥95% of tumor cells demonstrated classical apocrine morphology, including abundant granular eosinophilic cytoplasm, prominent nucleoli, and distinct cellular borders, in accordance with previously described criteria.

The androgen receptor (AR) status was assessed by immunohistochemistry (IHC) using the Dako antibody (clone AR441) and was defined as positive if ≥10% of tumor nuclei were stained. Additional IHC for luminal differentiation markers (FOXA1 and GATA3) was performed in a subset of TNAC tumors but not evaluated routinely in the NA-TNBC cohort.

Pathological complete response (pCR) was defined consistently as the absence of residual invasive carcinoma in both the breast and axillary lymph nodes (ypT0/is, ypN0).

Tumor-Infiltrating Lymphocytes (TILs) Assessment.

TILs were evaluated on stromal areas of H&E-stained sections in accordance with the International TILs Working Group (ITWG) 2014 recommendations [29]. A semi-quantitative scoring system was applied, in which tumors were categorized as:

- Score 0 (negative): absent or negligible stromal lymphocytic infiltration;
- Score 1 (mild): low-level stromal infiltration;
- Score 2 (moderate to high): dense stromal lymphocytic infiltration.

For analytic purposes, cases with moderate-to-high stromal infiltration (Score 2) were classified as TIL-positive, whereas tumors with Score 0 or 1 were considered TIL-negative. In selected cases, additional immunohistochemistry for CD8 and FOXP3 was performed to further characterize cytotoxic and regulatory T-cell subpopulations.

PD-L1 expression was assessed by IHC using the Dako 22C3 pharmDx assay, and results were reported according to the combined positive score (CPS) method to ensure consistency and reproducibility.

Ki-67 proliferation index was assessed by immunohistochemistry on pre-NACT core biopsy samples and post-NACT surgical specimens. The percentage of positively stained tumor nuclei was recorded by two experienced pathologists. Delta Ki-67 was calculated as the absolute difference between baseline and post-treatment Ki-67 values.

Cut-off values were determined based on survival analyses. Although receiver operating characteristic (ROC) curve analysis was performed to identify optimal thresholds, no consistent or clinically meaningful value could be established. Therefore, median values within the study cohort were adopted to dichotomize patients for subsequent analyses: baseline Ki-67 was classified as high when ≥30%, and delta Ki-67 was considered significant when >20%.

### 2.3. Treatment Protocol

All patients received anthracycline-based NACT regimens combined with a taxane (either docetaxel or paclitaxel). Among them, 52 patients additionally received carboplatin, based on clinical discretion. Importantly, none of the patients in our cohort were treated with dose-dense schedules; all received conventional dosing regimens. During the study period (2010–2022), immunotherapy was not available in our country; therefore, no patient received immune checkpoint inhibitors as part of their neoadjuvant treatment.

### 2.4. Statistical Analysis

All statistical analyses were conducted using IBM SPSS Statistics version 28.0. Categorical variables were compared using the Chi-square test, or Fisher’s exact test when subgroup sizes were small. Continuous variables were compared using Student’s *t*-test or Mann–Whitney U test, as appropriate.

Overall survival (OS) was estimated using the Kaplan–Meier method. Median follow-up duration was calculated using the reverse Kaplan–Meier method. Survival curves were compared using the log-rank test. Numbers at risk were displayed below Kaplan–Meier plots at pre-specified time points to provide clarity regarding patient attrition over time.

Multivariable Cox proportional hazards models were performed to identify independent prognostic factors for overall survival (OS). Results are reported as hazard ratios (HRs) with corresponding 95% confidence intervals (CIs) and *p* values, and proportional hazards assumptions were formally tested.

Logistic regression analyses were used to explore predictors of pathological complete response (pCR), and results are reported as odds ratios (ORs) with 95% CIs, correcting the mislabeling in earlier drafts.

A two-sided *p*-value < 0.05 was considered statistically significant for all analyses.

## 3. Results

### 3.1. Patient Characteristics

A total of 509 patients with locally advanced breast cancer who received NACT were initially screened. After excluding hormone receptor-positive (n = 293) and HER2-positive (n = 87) cases, 129 patients with confirmed TNBC remained eligible for analysis. Of these, 84 (65.1%) were classified as non-apocrine TNBC (NA-TNBC), and 45 (34.9%) were diagnosed with triple-negative apocrine carcinoma (TNAC) based on strict histological criteria.

The mean age at diagnosis was significantly higher in the TNAC group (58.3 ± 10.6 years) compared to the NA-TNBC group (52.7 ± 9.4 years, *p* = 0.01), consistent with the literature, which suggests a later onset of apocrine tumors. TNAC tumors were more likely to be histologically grade 2 (48.9% vs. 14.3%, *p* < 0.001), while NA-TNBC tumors were predominantly grade 3. Multifocality was also more frequent among TNAC cases (20% vs. 8.3%), though this difference was not statistically significant (*p* = 0.08).

AR positivity was observed in 64.4% (29/45) of TNAC cases, whereas all NA-TNBC cases were AR-negative by definition.

### 3.2. Treatment Characteristics and Chemotherapy Regimens

All patients received anthracycline-based NACT regimens combined with a taxane (either docetaxel or paclitaxel). Among them, 52 patients additionally received carboplatin, based on clinical discretion (52.4% of NA-TNBC patients and only 8.3% of TNAC patients). Importantly, none of the patients in our cohort were treated with dose-dense schedules; all received conventional dosing regimens. Among TNAC patients who received carboplatin (n = 4), 2 achieved pCR (50%), suggesting a potential benefit in select individuals. In contrast, pCR was observed in only 1 of 41 (2.4%) TNAC patients who did not receive carboplatin (Fisher’s exact test, *p* = 0.018). Although subgroup size was limited, this finding may warrant further investigation in prospective studies.

### 3.3. Post-NACT Surgery Details

In the entire study population, the rate of breast-conserving surgery (BCS) was 55%. When stratified by histologic subtype, BCS was significantly more frequent in the TNAC group compared with the non-TNAC group (75.6% vs. 44.0%, *p* < 0.001). Surgical margins were negative in all patients. Detailed information on surgical indication criteria and axillary management (SLNB, ALND, or RT) could not be reliably retrieved from the retrospective oncology files, which represents a limitation of our study.

This imbalance in surgical approach may also introduce potential confounding. Specifically, the higher frequency of BCS in the TNAC group may partly reflect smaller tumor burden, more favorable anatomical localization, or differential surgical decision-making, rather than being solely attributable to biological characteristics of apocrine carcinoma. Therefore, the observed association between histology and surgical type should be interpreted with caution.

Baseline tumor size and nodal status did not differ significantly between groups, with median tumor diameters of 3.4 cm (TNAC) and 3.7 cm (NA-TNBC). Axillary lymph node involvement at diagnosis was present in 46.7% of TNAC and 51.2% of NA-TNBC cases.

Clinicopathological characteristics of patients with apocrine differentiation are presented in Table 1 and Table 2.

### 3.4. Pathological Complete Response (pCR)

The overall pCR rate for the entire cohort was 22.4% (29/129). When stratified by histological subtype, TNAC patients had significantly lower pCR rates than NA-TNBC patients (6.6% vs. 30.9%, *p* = 0.002).

Univariate analysis revealed several factors significantly associated with pCR: tumor grade 3 (*p* < 0.01), high baseline Ki-67 (≥30%, *p* < 0.001), and delta Ki-67 > 20% (*p* < 0.001). Apocrine histology and AR positivity were associated with a lower likelihood of achieving pCR.

The median baseline Ki-67 index was 30% in TNAC and 70% in NA-TNBC (*p* < 0.001). Median post-treatment Ki-67 values were 10% for TNAC and 20% for NA-TNBC. Consequently, delta Ki-67 was significantly lower in TNAC patients (median: 16.2%) compared to NA-TNBC patients (median: 38.1%, *p* < 0.001).

Notably, 86% of patients who achieved pCR had delta Ki-67 values above the cohort median, reinforcing its predictive value. Logistic regression identified high tumor grade and delta Ki-67 > 20% as independent predictors of pCR (OR = 3.21, 95% CI 1.8–5.7, *p* < 0.001). Logistic regression analysis for predicting pCR is presented in Table 3.

### 3.5. Immune Microenvironment Analysis

TIL evaluation based on semi-quantitative scoring (0–2) demonstrated no statistically significant differences between subtypes. The proportion of tumors with moderate-to-high stromal infiltration (Score 2, defined as TIL-positive) was 11.1% in TNAC and 16.6% in non-apocrine TNBC (*p* > 0.05). Conversely, TIL-negative tumors (Score 0) were more frequent in non-apocrine TNBC (26.0%) compared with TNAC (8.8%), but this difference did not reach statistical significance.

PD-L1 positivity (CPS ≥ 10) was detected in 12.5% of TNAC Vs. 43.7% of non-apocrine TNBC, indicating a lower prevalence of PD-L1 expression in TNAC.

Taken together, the low frequency of TIL-positive tumors and the reduced rate of PD-L1 expression in TNAC suggest that this subtype exhibits features of an “immune-cold” phenotype. These results align with recent literature indicating that apocrine carcinomas generally harbor limited immunogenic activity and diminished sensitivity to immune checkpoint inhibition. However, given the retrospective design and limited subgroup sizes, these findings should be regarded as exploratory and hypothesis-generating.

### 3.6. Survival Outcomes

At a median follow-up of 32.5 months (range: 7.2–134.6 months), 20 patients (15.5%) had died, and 23 (17.8%) had experienced disease progression. Of the 45 TNAC patients, four deaths and nine progressions were recorded.

Kaplan–Meier survival analysis demonstrated a non-significant but favorable trend in 5-year OS for TNAC patients (86%) compared to NA-TNBC (78%, log-rank *p* = 0.12). Disease-free survival (DFS) also trended higher in TNAC, although median DFS was not reached in either group.

Among TNAC patients, AR-positive tumors had a higher 5-year OS (91%) compared to AR-negative tumors (82%), although this did not reach statistical significance (*p* = 0.18).

Patients achieving pCR had markedly superior OS and DFS compared to non-pCR counterparts, regardless of subtype (5-year OS: 95.8% vs. 72.6%, *p* < 0.001). Multivariate Cox regression confirmed pCR and earlier clinical stage at diagnosis as independent predictors of OS.

**Figure 1:**
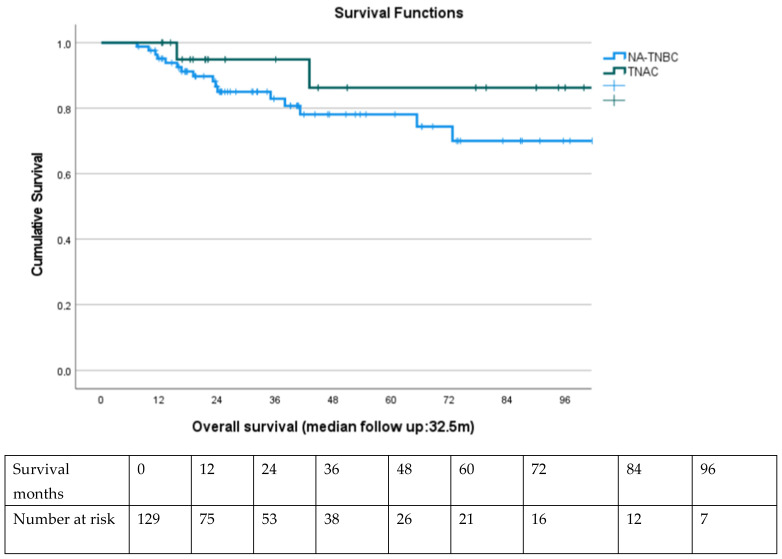
Kaplan–Meier curves showing the difference in survival between TNAC and NA-TNBC.

Kaplan–Meier curves are presented in Figure 1.

### 3.7. Subgroup Analysis

A post hoc exploratory analysis of the TNAC subgroup compared outcomes between patients who did and did not receive carboplatin. Although the rate of pCR appeared higher among those treated with carboplatin (50% vs. 2.4%, *p* = 0.018), the number of patients in this subgroup was very limited. Accordingly, this observation should be regarded as hypothesis-generating rather than definitive evidence and warrants confirmation in larger, prospective cohorts.

A post hoc analysis comparing TNAC patients with and without carboplatin

Furthermore, when stratified by AR status, AR-positive TNAC patients had numerically higher pCR rates (10.3%) compared to AR-negative TNAC patients (0%), although this difference was not statistically significant. This may reflect a biologically heterogeneous response profile even within TNAC, supporting the need for biomarker-driven treatment selection.

Cox Regression analysis that predicts overall survival is presented in Table 4.

## 4. Discussion

This study represents one of the most comprehensive comparative analyses of TNAC and NA-TNBC in the neoadjuvant setting. Our findings affirm that TNAC is a biologically and clinically distinct TNBC subtype, with unique histopathological features, treatment responses, and survival outcomes.

As anticipated, TNAC tumors were significantly less proliferative, demonstrated lower baseline Ki-67 levels, and had lower delta Ki-67 changes after NACT. These tumors also exhibited a dramatically lower pCR rate (6.6% vs. 30.9%) despite showing more favorable long-term survival outcomes. This paradox—poor initial treatment response but superior survival—highlights the inadequacy of applying traditional TNBC treatment paradigms uniformly across all histological subtypes.

### 4.1. Implications of Low Proliferative Activity and Delta Ki-67 in TNAC

Ki-67 is widely accepted as a surrogate of proliferation and chemosensitivity in breast cancer, particularly in TNBC. In our cohort, a delta Ki-67 greater than 20% was the strongest independent predictor of achieving pCR, consistent with prior reports emphasizing its predictive value across molecular subtypes [30,31]. Patients with delta Ki-67 > 20% were significantly more likely to achieve pCR, whereas those with persistently high post-NACT Ki-67 or delta Ki-67 ≤ 20% had markedly lower response rates.

Clinically, these findings may suggest a framework for tailoring treatment intensity. Treatment intensification may be warranted in patients with insufficient Ki-67 suppression (delta ≤ 20%), who are at higher risk of chemoresistance. Conversely, in patients demonstrating robust declines in Ki-67 (delta > 20%), treatment de-escalation strategies could be explored prospectively to minimize toxicity without compromising efficacy.

Of note, TNAC tumors consistently displayed lower baseline Ki-67 and less dynamic reduction post-NACT compared to non-apocrine TNBC, which correlated with poor chemotherapy response. This highlights the biological mismatch between standard cytotoxic regimens designed for rapidly dividing basal-like TNBC and the relatively indolent, luminal-like biology of TNAC.

### 4.2. Surgical Considerations in TNAC

Our findings demonstrated that BCS was significantly more frequent among TNAC patients compared with NA-TNBC (75.6% vs. 44.0%, *p* < 0.001), with negative surgical margins achieved in all cases. This observation raises an important clinical consideration: despite lower pCR rates, TNAC patients—particularly those with AR-positive/LAR-like biology—may be more suitable candidates for upfront surgery, given their relatively favorable tumor biology and surgical feasibility. The higher frequency of BCS in our TNAC cohort may, in part, reflect smaller tumor burden, favorable anatomical localization, or surgeon preference, rather than intrinsic histological differences. Nevertheless, the potential role of upfront surgery in TNAC warrants further exploration, especially regarding margin assessment and axillary management strategies (SLNB/ALND/RT).

Consistent with our results, recent evidence suggests that mastectomy is not necessarily superior to BCS in TNBC, provided that clear margins are achieved and multimodality treatment is applied [32]. This reinforces the need for individualized surgical decision-making in TNAC, balancing oncological safety with quality-of-life considerations.

### 4.3. AR Pathway: Prognostic and Therapeutic Significance

A defining feature of TNAC is its high prevalence of androgen receptor (AR) expression. In our cohort, 64.4% of TNAC tumors were AR-positive, consistent with previous reports indicating >90% AR positivity in apocrine carcinomas [14,15]. Interestingly, AR-positive TNAC patients demonstrated a trend toward improved 5-year OS compared to AR-negative cases (91% vs. 82%), suggesting a potential prognostic role for AR.

Biologically, AR activation drives a luminal transcriptional program (including FOXA1 and GATA3), resembling luminal A breast cancers. This hormonal circuitry provides a strong rationale for leveraging AR antagonists. Phase II trials in metastatic AR-positive TNBC have reported clinical benefit rates up to 39% with enzalutamide [33], highlighting the therapeutic feasibility of this approach.

In light of the limited chemosensitivity and consistently low delta Ki-67 values in AR-positive TNAC, AR blockade may represent a feasible alternative or adjunct strategy in the early-stage setting. While its role in the neoadjuvant or adjuvant context remains unexplored, incorporation of AR-targeted therapy could improve treatment tolerability and reduce unnecessary exposure to ineffective chemotherapy. Moreover, predictive biomarkers such as AR IHC scoring, nuclear FOXA1 co-expression, or AR-driven transcriptional signatures warrant further study to optimize patient selection.

### 4.4. The Case for Platinum in Selected TNAC Patients

Although the current consensus supports the use of platinum agents in TNBC, particularly in those with BRCA1/2 mutations or high homologous recombination deficiency (HRD), TNAC appears genomically distinct. As demonstrated in our study and others [22], TNAC exhibits a lower prevalence of BRCA mutations, reduced genomic instability, and diminished TMB—hallmarks associated with platinum sensitivity.

Yet, in our subgroup analysis, TNAC patients who received carboplatin showed a significantly higher pCR rate (50% vs. 2.4%), suggesting that a subset may still derive benefit. This raises the possibility that platinum agents could be selectively effective in TNAC patients harboring actionable PI3K pathway alterations or DNA repair defects outside the BRCA axis.

Future trials with integrated molecular profiling and biomarker stratification are essential to identify this responsive subset. Parallel efforts could explore the role of ATR inhibitors, PARP inhibitors in non-BRCA-mutated TNAC, or even synthetic lethal strategies tailored to the AR/PI3K interplay.

### 4.5. Immunological “Coldness” of TNAC and Emerging Strategies

One of the most compelling findings in this study was the stark contrast in the immune landscape between TNAC and NA-TNBC. TNAC tumors exhibited significantly lower stromal TILs, CD8+ infiltration, and PD-L1 expression—classifying them as “immune-cold” tumors. These characteristics may account for their limited response to immune checkpoint inhibitors, as demonstrated in major trials like KEYNOTE-522 and IMpassion130, where PD-L1 positivity was a critical determinant of benefit [20,21,22].

Epigenetic silencing of interferon response genes and antigen-presenting machinery has been proposed as a mechanism of immune evasion in TNAC [34]. Preclinical data indicate that HDAC inhibitors can restore MHC-I expression and enhance T cell infiltration in immune-desert tumors, including AR-positive TNBC models.

Combining epigenetic modulators with immune checkpoint inhibitors and AR blockade could “warm up” TNAC tumors and sensitize them to immunotherapy. STING agonists and CD73 inhibitors also represent novel avenues to boost immunogenicity in these tumors.

### 4.6. Microenvironmental Mechanisms and Exosome Biology in TNAC

Our data show a low prevalence of TIL-positive tumors and reduced PD-L1 expression in TNAC, consistent with an immune “cold” microenvironment and with diminished sensitivity to immune checkpoint inhibition in this subtype. In TNBC more broadly, exosomes orchestrate cross-talk among cancer cells, immune effectors, and stromal compartments, exerting context-dependent pro- and anti-tumor effects and shaping immune evasion [23].

For TNAC, systems-level approaches—network pharmacology integrated with transcriptomic and proteomic profiling—could identify AR-driven and PI3K/AKT/mTOR-linked nodes and map their interactions with the p53 axis [25]. Mechanistically, dysregulated microRNAs within the p53 pathway have emerged as key modulators of TNBC progression [27], and preclinical data suggest that natural compounds such as resveratrol may modulate these networks with potential therapeutic relevance (PMID: 35713130). While our clinical cohort was not designed to capture exosome cargo or miRNA circuitry, future TNAC-focused studies should incorporate liquid biopsy exosomal profiling, small-RNA/miRNA sequencing, and spatial multi-omics, alongside network-based analyses, to nominate actionable targets and rational combinations (e.g., AR blockade with pathway inhibitors or TME-modulating strategies).

### 4.7. Clinical Translation and Future Directions

Our study underlines the urgent need to reframe how TNAC is managed in both research and clinical settings. The lumping of TNAC into general TNBC trials has historically hindered the development of subtype-specific therapies. Dedicated clinical trials with TNAC-specific arms and biomarker-enriched cohorts are essential to refine treatment paradigms.

Furthermore, integration of genomic and transcriptomic profiling into diagnostic workflows may help accurately classify TNAC beyond morphology or AR IHC alone. High-throughput technologies, such as RNA sequencing or targeted NGS panels, could identify actionable mutations and immune profiles that guide individualized therapy.

Notably, the broader TNBC field is rapidly evolving. A systematic review and network meta-analysis by Cortes et al. (8) has demonstrated that the addition of immunotherapy to standard NACT significantly improves pCR and event-free survival in high-risk early-stage TNBC. Similarly, Leon-Ferre & Goetz [9] comprehensively summarized the expanding systemic therapy options—including immunotherapy, PARP inhibition, and antibody–drug conjugates—that have transformed TNBC treatment in recent years. These advances highlight the contrast with TNAC, which remains largely excluded from immunotherapy benefit due to its immune-cold biology, underscoring the need for alternative, biomarker-driven strategies in this rare subtype.

In clinical practice, the following steps may improve outcomes for TNAC patients:

1. Routine inclusion of AR and delta Ki-67 in pathology reporting.
2. Consideration of AR antagonists in both early-stage and metastatic settings.
3. Selective use of platinum agents based on molecular features.
4. Participation in biomarker-driven clinical trials exploring PI3K, mTOR, and epigenetic targets.
5. Avoidance of unnecessary chemotherapy in patients unlikely to benefit, as identified by low Ki-67, AR positivity, and immunological “cold” phenotype.

## 5. Conclusions

Triple-negative apocrine carcinoma (TNAC) represents a rare but biologically and clinically distinct subtype within the heterogeneous spectrum of triple-negative breast cancer (TNBC). Our study underscores the divergent behavior of TNAC compared to non-apocrine TNBC (NA-TNBC), particularly with respect to its low proliferative index, unique molecular features, immune landscape, and response to neoadjuvant chemotherapy.

Despite significantly lower pathological complete response (pCR) rates, TNAC patients demonstrated superior long-term survival, likely reflecting its intrinsically indolent biology. These findings support the emerging view that treatment efficacy in TNAC should not be gauged solely by pCR outcomes, but rather by a comprehensive assessment of molecular and immunologic context.

Importantly, we identified delta Ki-67 and high tumor grade as independent predictors of pCR, while AR positivity and apocrine histology, though negatively correlated with chemotherapy response, were associated with favorable survival outcomes. This supports the dual utility of AR as both a prognostic and potentially predictive biomarker.

Our results also emphasize the need for personalized treatment strategies in TNAC:

- AR-targeted therapy should be evaluated as an alternative or adjunct to cytotoxic chemotherapy.
- Selective use of platinum agents may be justified in biomarker-defined subsets.
- Immunotherapeutic approaches may require combination with epigenetic or PI3K pathway modulators to overcome intrinsic immune resistance.

The integration of TNAC-specific biomarkers such as AR status, Ki-67 dynamics, PI3K/mTOR mutations, and TIL density into routine diagnostic and therapeutic workflows could inform future biomarker-driven clinical trial designs.

In summary, treating TNAC as a biologically independent entity rather than subsuming it under the broader TNBC classification is critical to advancing precision oncology. Multimodal biomarker evaluation, molecular subtyping, and targeted therapeutic development hold the key to optimizing outcomes for this rare but impactful TNBC subset.

## 6. Limitations and Strengths of the Study

This study has several limitations that merit consideration. First, the retrospective and single-center design introduces inherent selection biases and limits the generalizability of the findings. In addition, the long study period (2010–2022) may have introduced era effects, as systemic therapy standards for TNBC evolved substantially during this timeframe. Second, the relatively small number of patients with triple-negative apocrine carcinoma (TNAC), particularly those who received carboplatin, restricted the statistical power of subgroup analyses. In particular, conclusions regarding chemotherapy efficacy in AR-positive versus AR-negative TNAC must be interpreted with caution due to the limited sample size. Third, genomic profiling, including BRCA mutation status, PI3K/AKT/mTOR alterations, and homologous recombination deficiency (HRD), was not routinely performed, which precluded deeper molecular correlation. Additionally, immune profiling was limited to a subset of patients and did not incorporate PD-L1 scoring by standardized platforms such as SP142 or 22C3 clones. Furthermore, treatment decisions (e.g., the use of platinum, immunotherapy, or surgical type) were not randomized but rather determined by physician discretion, which may confound outcomes.

Despite these limitations, the study possesses important strengths. It is one of the largest dedicated cohorts to explore clinical, pathological, and immunological features of TNAC compared to non-apocrine TNBC (NA-TNBC) in a real-world neoadjuvant setting. All cases were centrally reviewed by experienced breast pathologists, ensuring accurate histologic and immunohistochemical classification. Moreover, the inclusion of dynamic proliferation markers (delta Ki-67), AR expression, and TIL quantification enhances the translational relevance of the findings. Finally, the study provides hypothesis-generating insights that may inform future prospective trials tailored to TNAC biology.

## Figures and Tables

**Table 1:** Categorical variables of patients.

Categorical Variables of Patients	Apocrine Differentiation			
		No n (%)	Yes n (%)	*p* Value
Gender				<0.001
	female	84 (68.3)	39 (31.7)	
	male	0 (0)	6 (100)	
Age at diagnosis				<0.001
	≤60	75 (79.8)	19 (20.2)	
	>60	9 (25.7)	26 (74.3)	
ECOG-PS				0.002
	0	76 (71)	31 (29)	
	1	8 (36.4)	14 (63.6)	
Menopause status				<0.001
	premenopause	51 (87.9)	7 (12.1)	
	postmenopause	33 (46.5)	38 (53.5)	

Abbreviations: ECOG-PS: Eastern Cooperative Oncology Group-Performance Status.

**Table 2:** Categorical variables of tumor.

Categorical Variables of Tumor	Apocrine Differentiation			
		No n (%)	Yes n (%)	*p* Value
T stage at diagnosis				0.005
	1	16 (47.1)	18 (52.9)	
	2	44 (65.7)	23 (34.3)	
	3	15 (100)	0 (0)	
	4	9 (69.2)	4 (30.8)	
N stage at diagnosis				<0.001
	0	0 (0)	12 (100)	
	1	48 (63.2)	28 (36.8)	
	2	22 (81.5)	5 (18.5)	
	3	14 (100)	0 (0)	
Clinical Stage				<0.001
	2	37 (48.1)	40 (51.9)	
	3	47 (90.4)	5 (9.6)	
Grade				<0.001
	<3	23 (44.2)	29 (55.8)	
	≥3	61 (79.2)	16 (20.8)	
Pathological Response				0.002
	pCR	26 (89.7)	3 (10.3)	
	Non-pCR	58 (58)	42 (42)	
Median ∆ ki67				<0.001
	≤20	32 (47.8)	35 (52.2)	
	>20	52 (83.9)	10 (16.1)	
TIL score				0.046
	Score 0 (negative)	10 (76.9)	3 (23.1)	
	Score 1 (low)	12 (92.3)	1 (7.7)	
	Score 2 (moderate and high)	14 (73.7)	5 (26.3)	
	unknown	48 (57.1)	36 (42.9)	

Abbreviations: ∆ ki67: preop ki67–postop ki67 pCR: pathological complete response TIL: tumor-infiltrating lymphocyte.

**Table 3:** Logistic regression analysis predicting pathological complete response.

	Univariate Analysis				Multivariate Analysis		
Categorical Variables		*p* Value	OR	%95 CI	*p* Value	OR	%95 CI
Age at diagnosis ≤60 >60		**0.03**	4.07	1.14–14.47	0.49	1.71	0.36–8.01
ECOG-PS 0 1		0.11	3.37	0.74–15.39			
Menopause status premenopause postmenopause		0.21	1.70	0.73–3.90			
Grade ≤2 >2		**0.002**	5.76	1.87–17.78	**0.02**	3.91	1.19–12.82
	≤2						
	>2						
Apocrine differentiation No Yes		**0.004**	6.27	1.78–22.11	0.24	2.31	0.57–9.39
Androgen receptor expression Positive Negative		**0.03**	4.99	1.11–22.43	0.53	2.33	0.16–33.11
Median ∆ ki67 ≤20 >20		**<0.001**	10.64	3.43–32.97	**<0.001**	8.43	2.66–26.73
Median ki67 ≤60 >60		0.12	1.92	0.83–4.43			
Clinical Stage 2 3		0.32	1.52	0.66–3.50			
Clinical Lymph Node Status Positive Negative		0.09	0.96	0.76–4.46			

Abbreviations: ECOG-PS: Eastern Cooperative Oncology Group-Performance Status; pCR: pathological complete response, OR: odds ratio.

**Table 4:** Cox regression analysis predicting overall survival.

	Univariate Analysis				Multivariate Analysis		
Categorical Variables		*p* Value	HR	%95 CI	*p* Value	HR	%95 CI
Age at diagnosis ≤60 >60		0.48	1.38	0.55–3.47	0.06	1.62	0.42–6.18
ECOG-PS 0 1		0.70	1.26	0.37–4.33			
Menopause status premenopause postmenopause		0.89	0.94	0.39–2.28			
Grade ≤2 >2		0.46	1.43	0.55–3.73			
Apocrine differentiation No Yes		0.04	2.23	0.77–6.99	0.06	1.62	0.42–6.18
Androgen receptor expression Positive Negative		0.24	0.42	0.97–1.81			
Median ∆ ki67 ≤20 >20		0.22	1.75	0.71–4.29			
Median ki67 ≤60 >60		0.19	1.79	0.73–4.36			
Clinical Stage 2 3		0.005	4.31	1.56–11.87	0.008	2.24	0.08–6.88
Clinical Lymph Node Status Positive Negative		0.03	4.16	0.68–4.40	0.98	0.64	0.65–1.24
Pathological Response pCR Non-pCR		0.02	2.48	0.57–10.71	0.01	2.28	0.63–10.77

Abbreviations: ECOG-PS: Eastern Cooperative Oncology Group-Performance Status; pCR: pathological complete response. HR: hazard ratio.

## Data Availability

The data supporting the findings of this study are available from the corresponding author upon reasonable request. Due to institutional regulations and patient confidentiality agreements, the raw data are not publicly available. Statistical analyses were performed using IBM SPSS Statistics version 28.0. No custom code was generated.
